# Effectiveness of Colonoscopy Screening in Identifying Colorectal Cancer in Young Patients: A Retrospective Cohort Study in a Single Saudi Institution

**DOI:** 10.7759/cureus.84013

**Published:** 2025-05-13

**Authors:** Alaa Magdy, Mohamed Youssef, Heba Jabr, Lujain Alessawi, Taif Alharbi, Reem Almujil, Sara Hefni, Shokran Sulaimani, Alwaleed Alabdali, Abdulrahman Alotaibi

**Affiliations:** 1 Clinical Sciences, Fakeeh College for Medical Sciences, Jeddah, SAU; 2 General Surgery, University of Jeddah, Jeddah, SAU; 3 General Surgery, Doctor Soliman Fakeeh Hospital, Jeddah, SAU

**Keywords:** colorectal cancer, epidemiology, sigmoidoscopy, surveillance epidemiology and end results (seer), young population

## Abstract

Background

Globally, the incidence of colorectal cancer (CRC) is rising, with a notable increase seen in younger people. When detected early, CRC is a relatively curable illness with a 90% survival rate. Only those over 50 are recommended to be screened for colorectal cancer in those at average risk. The purpose of this study is to determine the likelihood of cancer in the community and to examine the dubious clinical signs that lead to colonoscopy in younger patients.

Methods

This retrospective cohort study analyzed medical records of patients aged ≤35 years who underwent colonoscopy between January 2021 and December 2024 at Doctor Soliman Fakeeh Hospital and had no prior rationale for early bowel cancer screening.

Results

A total of 312 participants had colonoscopies during the research period. Of them, 293 (93.9%) had symptoms before colonoscopy, such as abdominal pain (9.9%), symptomatic anemia (7.3%), abnormal bowel habits (22.9%), and rectal bleeding (53.8%). Three individuals (0.96%) with invasive cancer were identified to have stage III disease based on the histological analysis.

Conclusion

The incidence of CRC in a community that is thought to be low-risk is about 1%. Younger patients typically have a low suspicion of cancer, therefore delays in their inquiry could lead to a more advanced diagnosis than initially assumed. When a young patient shows any warning signs, it is therefore recommended that they be evaluated immediately.

## Introduction

Colorectal cancer (CRC) is the second leading cause of cancer-related fatalities and the third most common malignancy in both sexes [1]. In the majority of developed countries, the incidence of CRC has reached a stable level [2]. Since the mid-1980s, the incidence of CRC has been decreasing in patients over 50 years of age. This may be due to the introduction of screening tests such as colonoscopies and fecal occult blood tests, which make it possible to easily identify and remove adenomatous precancerous polyps. Additionally, there has been an increase in public awareness of the factors that increase the likelihood of developing CRC [3-5]. Between 1990 and 2019, the incidence of early-onset CRC increased from 4.2 to 100,000 to 6.7 to 100,000 among people aged 20 to 49, compared to those aged 50 to 74 years old. This trend is noticeable among younger age groups [6]. According to the national standards [7], the vast majority of young people fall below the inclusion limits for continuing bowel cancer screening. According to some research, younger people with CRC are more likely to be diagnosed at an advanced stage [8]. A study using the Surveillance, Epidemiology, and End Results (SEER) database found that younger patients had a higher frequency of stage III and IV disease than older patients, and the difference was statistically significant [9].

Clinical outcomes in young CRC patients have been reported in varying degrees in studies. A study found that younger patients with more advanced CRC had a higher risk of long-term mortality than older patients [10]. In Saudi Arabia (SA), CRC is the most prevalent cancer in men (13% of all cancers) and the second most prevalent cancer in women (9%), and a total of nearly 1,200 cases were reported in 2011 [11]. Of particular note, the age of diagnosis (55-58 years) is roughly 12-15 years lower than that of Western populations [12-14]. Compared to the Western countries, Saudi Arabia has an approximately three to four-fold reduced age standardized incidence of CRC, although the incidence is increasing relatively rapidly [15], presumably due to increasing Westernization of the diet.

## Materials and methods

Study design, setting, and participants

This retrospective cohort study was conducted at the Dr. Soliman Fakeeh Hospital (DSFH), a referral center in Jeddah, Saudi Arabia, from January 2021 to December 2024. The objective was to determine the frequency and characteristics of CRC among patients aged 35 years or younger presenting for colonoscopy. The hospital's electronic medical records were used to retrospectively screen patients who had colonoscopies throughout the study period and who satisfied the inclusion requirements. Those under 35 who had no clinical cause for an early colonoscopy screening for bowel cancer were eligible participants. Participants were not enrolled if they had a personal history of colorectal malignancy or colonic polyps, known hereditary syndromes (like serrated polyposis syndrome), inflammatory bowel diseases (IBD), or a family history of high-risk CRC. A high-risk family history entailed a first-degree relative who had been diagnosed with CRC at or before age 55, two first-degree relatives who had CRC, or a mixture of one first-degree and one second-degree relative with CRC on the same side. The primary end point was the risk of CRC among the selected patients, and the secondary end points were the detection of other pathologies.

Sample size and sampling technique

The records of 312 patients were screened on an availability and eligibility basis within the provided time limit. All eligible available cases were utilized, and no sample size calculation was required due to the nature of retrospective sampling.

Data collection tools and procedures

Data were obtained from patient charts and electronic medical records with the use of a standardized data collection form. Data collected included demographics (age, sex), colonoscopy indication, endoscopic findings, and histopathological reports. Indications for colonoscopy were classified into five pre-defined categories: rectal bleeding, change in bowel habits (e.g., diarrhea, constipation), abdominal pain, symptomatic anemia. Histopathology was performed to confirm the diagnosis of CRC or an alternative pathology. The stage of cancer at diagnosis was obtained from biopsy and surgical histology reports where available.

Ethical considerations

The data handling in this study complied with applicable ethical standards. The study protocol was reviewed and approved by the Fakeeh College for Medical Sciences Institutional Review Board (IRB) in Jeddah (approval number: 612/IRB/2024). This ensured adherence to institutional and international ethical guidelines, including implied compliance with principles similar to the Declaration of Helsinki.

All patient data were anonymized and handled confidentially, protecting participant privacy. As a retrospective study utilizing existing medical records, patient consent was exempted by the IRB. This aligns with ethical standards for retrospective research where anonymized data are used without direct patient interaction. All authors declared no conflicts of interest and confirmed ethical compliance in the "Disclosures" section, including IRB approval and appropriate consent procedures.

Data management and analysis

Data were entered and analyzed using IBM SPSS Statistics for Windows, Version 22 (Released 2013; IBM Corp., Armonk, New York, United States). Descriptive statistics were used in reporting demographic and clinical information, categorical variables were presented in frequencies and percentages. The proportion of CRC cases confirmed by histopathology (i.e., biopsy) among all colonoscopies performed was also recorded.

## Results

Three hundred twelve patients underwent colonoscopy during the designated study period. Male patients constituted 51.9% of the sample size. Among the participants, the most prevalent body mass index (BMI) category was overweight (42.3%). Of the 312 patients, 23 (7.4%) were identified as current smokers, 33 (10.6%) had diabetes, and 39 (12.5%) had hypertension. In terms of symptomatology, 293 (93.9%) patients exhibited symptoms before the procedure, including rectal bleeding (53.8%), altered bowel habits (22.9%), abdominal pain (9.9%), and symptomatic anemia (7.3%) (Table 1). 

**Table 1:** Demographic and clinical characteristics of the young adult patients included in the study (n=312)

Variable		N	Percentage (%)
Gender	Male	162	51.9%
	Female	150	48.1%
BMI (kg/m^2^)	Underweight (<18.5)	11	3.5%
	Normal (18.5–24.9)	102	32.7%
	Overweight (25.0–29.9)	132	42.3%
	Obese (≥30.0)	67	21.5%
Smoking		23	7.4%
Diabetes mellitus		33	10.6%
Hypertension		39	12.5%
Symptom	Rectal bleeding	168	53.8%
	Altered bowel habits	71	22.9%
	Abdominal pain	31	9.9%
	Symptomatic anemia	23	7.3%
	Asymptomatic/other	19	6.1%

Mechanical bowel preparations were reported in 276 patients (88.46%) with the ability to perform cecum intubation in 288 patients (92.3%) (Table 2). 

**Table 2:** Colonoscopy performance, adverse events, and diagnostic outcomes in the study population (n=312)

Section	Variable	N	Percentage (%)
Colonoscopy performance	Good mechanical bowel preparation	276	88.5%
	Successful cecum intubation	288	92.3%
Adverse events	Major bleeding (conservative treatment)	1	0.3%
Primary endpoint	Colorectal carcinoma detection	3	0.96%
Secondary endpoint	Adenoma detection	124	39.7%
	Inflammatory bowel disease (IBD) detection	8	2.6%
Diagnostic findings	Patients with positive findings	132	42.3%
	Patients with no findings	180	57.7%

**Figure 1:**
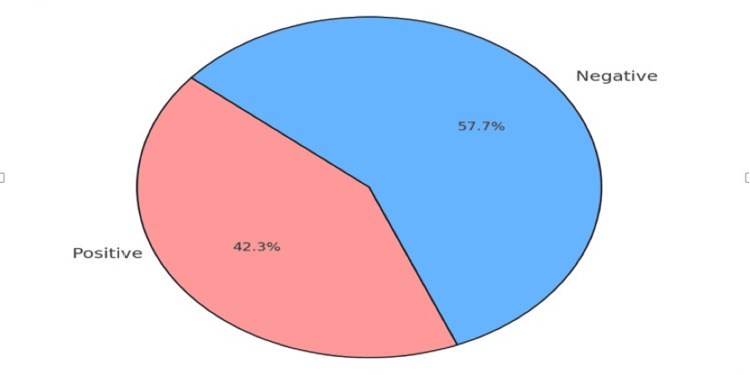
Colonoscopy findings in the study population (n=312)

Only one patient complained of post-procedural bleeding that was treated conservatively (Table 2). Biopsies were performed in 148 of the 312 cases (47.4%), with an overall adenoma detection rate of 39.7%. Newly diagnosed inflammatory bowel disease was identified in eight cases (2.6%). Additionally, three cases of invasive adenocarcinoma were detected. These patients had experienced symptoms for more than eight weeks before diagnosis, with primary indications for colonoscopy being rectal bleeding and symptomatic anemia. Further investigations for these cases classified them as stage III disease in recto-sigmoid colon (Table 2). Colonoscopy was able to detect positive findings in 132 (42.3%) patients (Table 2, Figure 1).

## Discussion

The prevalence of CRC among the Saudi population is rising swiftly, making it the most common cancer type in males. The mean age of diagnosis for Saudi patients is roughly 15 years younger than that of Western patients with CRC, at 55 and 70 years, respectively [12,13]. The aim of this study was, therefore, to establish whether early screening of symptomatic patients could help us identify and diagnose CRC in these patients. In this retrospective study, we revealed that young patients diagnosed with CRC present with symptoms at the time of diagnosis. This aligns with the results from a study by Dozois et al., which was the most extensive single-institution cohort study on early-onset CRC at that time [8]. This study analyzed records of patients aged 50 or younger with a primary CRC diagnosis, excluding those with IBD, hereditary polyposis syndromes, or known genetic risk factors. Consistent with our results, they reported that 81% of patients were symptomatic at diagnosis, and all had tumors in the left colon or rectum. In contrast, another Saudi study reported that right-sided tumors were the most common among young CRC patients [16]. 

A significant proportion of CRC (68%) presented with stage III or IV disease. The stage distribution reported by Saudi cancer registry 2008 (stage III 38.8%, stage IV 29.2%) [17] was matched to that displayed in our study. It was noteworthy that advanced stages (stages III and IV) were more frequent in older compared to younger Saudi patients in the pooled data from the Saudi cancer registry between 2001 and 2006 [18]. Thus, our study coincides with a shift in the disease pattern among young CRC patients in recent years, as most are now presenting with advanced stages. Our research focused on patients aged 35 years or younger. A retrospective 30-year study by Fu et al. identified 35 years as an appropriate age threshold for defining early-onset CRC [19] by using a survival analysis. The study findings also highlighted a poorer prognosis among patients ≤35, attributed to the higher frequency of advanced-stage cancers, likely due to diagnostic delays caused by a low clinical suspicion of cancer in younger individuals [7]. This underscores the importance of early evaluation in young patients who exhibit warning signs and supports our call for heightened awareness and proactive symptom investigation in this population. 

The variation in clinical presentation between early-onset and older adult-onset CRC offers valuable insights into differences in symptomatology and disease progression across age groups. Abdominal pain was more frequently reported among younger patients, aligning with existing literature and indicating that early-onset CRC is often characterized by more acute symptomatology at the time of diagnosis [5,20]. Given that older patients are more likely to develop proximal colon cancers, flexible sigmoidoscopy may be a more efficient initial diagnostic tool for symptomatic younger patients than waiting for a full colonoscopy. In our study, three CRC cases involved obstructive tumors in the recto-sigmoid area, which could likely have been identified using flexible sigmoidoscopy. This method is widely accessible, cost-effective, and carries fewer risks. A randomized trial funded by the National Cancer Institute showed that flexible sigmoidoscopy significantly reduced both CRC incidence and mortality in individuals aged 55 to 74 [21]. Similarly, a UK-based randomized controlled trial confirmed that flexible sigmoidoscopy is a safe and effective long-term preventive screening tool [22]. In light of institutional limitations and long colonoscopy wait times, offering flexible sigmoidoscopy in clinical settings could provide significant benefits for younger patients.

Our results also align with a 2014 study utilizing the California Cancer Registry, which analyzed 231,544 CRC cases between 1988 and 2009 with a focus on younger adults [23]. The study confirmed that although CRC incidence is still lower in younger individuals compared to those over 50, it is steadily increasing in this demographic, contrary to declining rates among those eligible for screening programs. The success of CRC screening in adults aged 50 and above in reducing incidence and mortality has led to discussions about lowering the screening age [24]. In response, the American Cancer Society updated its guidelines, recommending routine CRC screening from the age of 45 years for individuals at average-risk [4]. This change highlights a disparity between Saudi Arabia and other international counterparts regarding efforts to reduce CRC-related morbidity and mortality. Nonetheless, Saudi Arabia has committed to expanding its screening program, aiming for full implementation by 2030. This expansion marks a departure from previous screening intervals and aligns with the current recommendations of the Saudi Health Council. This study was conducted during the early phase of Saudi Arabia’s national screening expansion efforts (2021-2024) to evaluate the effectiveness of colonoscopy in young symptomatic patients. The limitation of this study include the small sample size and being a single-center retrospective study.

## Conclusions

This retrospective cohort study highlights critical trends in CRC among young Saudi patients under 35 years of age. Despite being categorized as low-risk, approximately 1% of screened individuals were diagnosed with CRC, all presented with advanced (stage III) disease. These findings underscore the clinical challenge of delayed diagnosis in younger populations, where low suspicion of malignancy often leads to prolonged symptom duration before intervention.

The predominance of rectal bleeding and symptomatic anemia as primary indicators for colonoscopy suggests that these symptoms should prompt urgent evaluation in young patients. Flexible sigmoidoscopy, a less resource-intensive tool, may serve as a practical first-line diagnostic measure for recto-sigmoid tumors, particularly in settings with limited colonoscopy availability. While Saudi Arabia’s planned nationwide screening expansion by 2030 aligns with global efforts to reduce CRC burden, this study emphasizes the need for tailored strategies targeting symptomatic young adults. Future research should explore longitudinal outcomes and cost-effectiveness of early screening protocols in similar populations to optimize detection and survival rates.
